# Effects of six months of weekly supervision on using Prolonged Exposure Therapy for PTSD treatment in Ukraine

**DOI:** 10.21203/rs.3.rs-7567378/v1

**Published:** 2026-02-05

**Authors:** Tetiana Nickelsen, Gregory Muller, Shaunna Clark, Gahl Liberzon, Oleksandr Bordiuzhenko, Marcia Ory, Israel Liberzon

**Affiliations:** 1 Department of Psychiatry & Behavioral Sciences, Texas A&M University, College Station, Texas, USA; 2 Department of Psychiatry & Behavioral Sciences, University of Texas, Austin, Texas, USA; 3 School of Public Health, Texas A&M University, College Station, Texas, USA; 4 School of Psychological Sciences, University of California Irvine, Irvine, California, USA

**Keywords:** Trauma Treatment, PTSD, clinical supervision, Ukraine, implementation, global mental health, therapist confidence, intervention, adaptation, conflict-affected settings, remote training

## Abstract

**Background:**

Brief training can introduce clinicians to evidence-based psychotherapies, but ongoing supervision is often critical for successful implementation. This need is amplified in global mental health, where linguistic, cultural, and systemic barriers complicate the delivery of trauma-focused treatments such as Prolonged Exposure (PE) therapy for Post Traumatic Stress Disorder (PTSD). Following the onset of the full-scale war in Ukraine, we implemented a PE training and remote supervision initiative for Ukrainian psychiatrists and psychologists.

**Objective:**

This study evaluated the effects of six months of weekly remote supervision on clinicians’ confidence in implementing PE and examined whether supervision attendance and PE case initiation predicted post-supervision confidence. We also assessed changes in perceived barriers to implementation.

**Methods:**

Ukrainian clinicians (n=41) were trained in PE during a five-day in-country workshop. Assessments included 29 clinicians who joined six months of weekly online supervision with U.S.-based PE trainers. Confidence in delivering PE, was measured pre- and post-supervision using a Likert-scale self-report item. Linear regressions tested the predictive value of supervision attendance and PE case initiation on post-training confidence. Qualitative thematic analysis was applied to open-ended responses describing perceived implementation barriers before and after supervision.

**Results:**

Confidence in PE implementation significantly increased from pre- to post-supervision (p < .05). Both the number of supervision sessions attended and the number of PE cases initiated were significant independent predictors of post-supervision confidence (p < .05) and together explained over 30% of the variance. Thematic analysis revealed a shift in perceived barriers from structural and educational limitations to client-centered and logistical challenges following supervised practice.

**Conclusions:**

Remote supervision, despite language, time zone, and wartime obstacles, effectively enhanced clinicians’ confidence and understanding of implementation barriers. These findings demonstrate the feasibility and value of structured, fidelity-focused supervision in crisis settings and support its role in scaling trauma-focused care globally.

## Introduction:

Psychotherapeutic techniques can be effectively taught through reasonably short intensive training sessions (Beidas & Kendall, 2010) however, the successful implementation of those techniques is more challenging and depends on multiple factors (Finley et al., 2015). Among these factors, a key element that has been proposed by some researchers (Lyon et al., 2019) is ongoing supervision (Milne et al., 2011).

Ongoing supervision is commonly defined as a collaborative relationship between a more experienced clinician and a trainee that involves regular meetings focused on developing clinical skills, ensuring treatment fidelity, and promoting professional growth through case review, feedback, and guidance (Bernard & Goodyear, 2013).

Several distinct supervision models have emerged in clinical practice. Individual supervision, the traditional one-on-one format, allows for personalized attention and in-depth case conceptualization (Watkins Jr, 2014). Group supervision, involving one supervisor and multiple trainees, offers the advantages of peer learning and diverse perspectives while being more resource-efficient (Borders, 1991). Peer supervision, a more collaborative approach where clinicians of similar experience levels provide feedback to each other, promotes autonomy and reduces hierarchical barriers (Bernard & Goodyear, 2019). More recently, technology-mediated supervision through videoconferencing has gained prominence, particularly in geographically dispersed or crisis contexts, offering flexibility while maintaining essential supervisory elements (Rousmaniere et al., 2016). Additionally, competency-based supervision focuses specifically on developing measurable clinical skills rather than broader professional development (Falender & Shafranske, 2012).

The frequency, duration, and structure of supervision also vary considerably across training contexts. While weekly supervision is common, biweekly or monthly arrangements may be employed, particularly for more experienced trainees (Milne, 2009). The intensity of supervision can range from brief check-ins to extended sessions focused on detailed case review and skill development (Milne & Watkins Jr, 2014).

Various evidence-based approaches, such as Cognitive Therapy, Prolonged Exposure, Motivational Therapy, and Short Psychodynamic Psychotherapy, may each present unique supervision challenges and requirements due to their distinct theoretical frameworks, technical procedures, and implementation contexts (Falender & Shafranske, 2012). The relationship between different therapeutic techniques, the nature of supervision, and clinician attitudes toward and adoption of evidence-based trauma care remains an area of inquiry. Specifically, little is known about how clinical supervision affects therapist’s confidence in engaging in, and adoption of, Prolonged Exposure therapy for recent PTSD. This question is particularly salient in situations that require large scale training in evidence-based therapy, and especially if supervision is performed when the supervision language (English) is not a “mother tongue” of prospective trainees.

In this paper, we explore the impact of a six-month program of weekly Prolonged Exposure supervision, provided virtually by PE-certified trainers in the U.S. to Ukrainian therapists who were actively involved in providing trauma therapy services. We hypothesized that supervision would improve our trainees’ confidence in engaging in PE, one of the key factors in Evidence-Based Care (EBC) delivery, and thus measured the increase in confidence from pre- to- post supervision. We identified two readily modifiable factors that might contribute to more effective supervision outcome measured as “therapist confidence” at the end of supervision sessions. These were regular attendance in supervision sessions and number of PE cases that our trainee initiated during the six months of supervision. While those factors are likely not fully independent of each other, we assumed each one of them added unique variance to the final confidence, and each one of them can be addressed separately during the supervision period.

We hypothesized that regular supervision (number of sessions attended) and direct experience in delivering PE (new cases initiated) would enhance clinicians’ confidence, change the original perception of barriers, and support the successful adoption of PE in a new clinical setting.

## Methods:

### General procedure description:

After a five-day in-person workshop in Ukraine (Nickelsen et al., 2025) that included 16 hours of PE training (see content of training in Table 1), the scheduling of PE supervision was coordinated with the two PE trainers. Supervision was conducted with the goal of enhancing the trainees’ knowledge and clinical skills. Confidence ratings (see below) were collected as part of an internal QA/QC process. Following the completion of supervision, a secondary analysis was planned, and the IRB process was initiated. Subsequently, IRB approval and an exemption from informed consent were obtained (Division of Research, Human Research Protection Program, Texas A&M University #STUDY2024–0438). Supervision included a total of 24 weekly sessions, with the average number of sessions attended being seven. The structure of supervisory appointments included a 60-minute virtual meeting hosted via the Zoom Communications telehealth platform, across an 8-hour time zone differential. These supervision sessions were conducted in English. Typical sessions had a student-teacher ratio of five supervisees to two PE trainers. Meeting agendas within the early stages of PE supervision sought to further consolidate the Ukrainian supervisees’ conceptual understanding of the PE content was taught during the initial five-day workshop. Common topics included reviewing PE foundational psychoeducation, mechanisms of change, and strategies for dropout prevention. Early supervision also worked to assist the supervisees in identifying and acquiring appropriate PE clinical cases to begin developing their expertise. Once clinical cases were acquired, supervisees were supported in navigating case details and related challenges. Common challenges included management of treatment-interfering behaviors (e.g., poor attendance or homework completion) or comorbid conditions (e.g., substance abuse, head injuries). Session activities gradually increased in complexity as the supervisory experience continued. Later supervisory sessions more heavily emphasized PE protocol adherence. Supervisees’ questions also gradually began to reflect a more advanced understanding of PTSD etiology and the PE treatment process.

### Variable selection:

We selected four commonly used indices of supervision effectiveness to assess the effects of supervision we provided:

1. Therapist confidence: Therapist confidence represents a critical subjective measure of supervision effectiveness, particularly in trauma-focused therapies like Prolonged Exposure (PE). This construct encompasses therapists’ beliefs in their ability to implement specific techniques correctly, manage challenging clinical situations, and achieve positive outcomes with clients. Confidence assessment typically involves self-report measures using Likert scales that evaluate comfort with specific PE components (e.g., imaginal exposure, in vivo exposure) and overall treatment delivery (Shapiro et al., 2021).
2. Attendance (number of sessions): Regular participation in supervision was conceptualized as a behavioral indicator of trainee engagement and exposure to learning opportunities. Previous literature emphasizes the importance of consistent supervisory contact for skill consolidation, troubleshooting, and emotional support (Milne, 2009; Watkins Jr, 2014).
3. Caseload: Caseload measures provide objective data about the volume and completion rates of cases treated using the supervised therapy approach. These metrics typically include the number of clients assessed for treatment suitability, cases initiated, sessions delivered, and treatment completions (Dennhag & Armelius, 2012).
4. Qualitative feedback: Thematic analysis of trainees’ reported experiences and perceived barriers to implementation (Rakovshik et al., 2016).

### Data collection:

Trainees completed pre-supervision questionnaires prior to the start of the first supervision session. The same set of surveys was administered as a post-assessment immediately following the final supervision. Participants were given sufficient time to complete the questionnaires independently, and confidentiality of their responses was ensured. All surveys were distributed via Google Forms and completed in English.

The pre- and post-assessment questionnaires collected information on trainees’ background, profession, years of experience, and geographic region. To assess confidence in delivering PE therapy, we included the question: *“How confident do you feel in delivering Prolonged Exposure (PE) therapy?”* rated on a 5-point Likert scale: *Not at all confident, Slightly confident, Moderately confident, Very confident, Extremely confident*.

The number of supervision sessions attended was tracked by a research assistant. Participants self-reported the number of PE cases they had initiated.

To assess changes in the perception of barriers before and after attending supervision, we included the following open-ended question: “What do you see as the greatest barriers to implementing PE therapy? Please list up to three barriers.”

## Analysis:

We conducted all statistical analyses using IBM SPSS Statistics Version 29.0.1.1(244) (IBM Corp., Armonk, NY). Descriptive statistics including frequencies, percentages, means, and standard deviations were used to summarize participant demographics and supervision engagement.

To assess whether participation in supervision improved trainees’ confidence in delivering PE interventions, we compared self-reported confidence levels before the first supervision session and after six months of supervision using a one-tailed Wilcoxon signed-ranks test, with statistical significance set at *p* < .05.

We then fit a series of linear regression models to test the individual and combined effects of supervision session adherence and caseload initiation on post-supervision confidence in delivering PE. To test the individual effects, we regressed post-supervision confidence on the number of supervision sessions attended (Model 1) and the number of new PE cases initiated during supervision (Model 2) separately. To test the combined effect, we then included both the number of supervision sessions attended and number of new PE cases initiated as predictors of post-supervision confidence (Model 3). All models included pre-supervision confidence to control for baseline levels of confidence in delivering PE.

Finally, we employed thematic qualitative analysis (Braun & Clarke, 2006) to explore changes in the perception of barriers to delivering EBC among trainees before and after six months of supervised clinical practice using PE therapy. This analysis focused specifically on responses to the open-ended question: *“What do you see as the greatest barriers to implementing evidence-based practice in trauma care in Ukraine?”* Participants were asked to list up to three perceived barriers both prior to the training workshop and again after completing a six-month period of delivering PE under supervision. Thematic analysis followed Braun and Clarke’s six-phase framework: (1) familiarization with the data, (2) generation of initial codes, (3) search for themes, (4) review of themes, (5) definition and naming of themes, and (6) writing up the results. Two raters were involved in analysis; all responses were reviewed by both raters, and agreement was reached. Themes were identified through an inductive, data-driven process and categorized based on recurring patterns. Each response was coded, and the frequency of each theme was quantified to assess its relative prevalence. To ensure focus on the influence of clinical supervision, the analysis included only responses from trainees who had completed the full cycle of supervision. Responses from those who attended the training but did not participate in supervision were excluded. The comparison of pre- and post-supervision responses allowed for the identification of shifts in perceived barriers over time. Themes were visualized using a bar chart to illustrate both the overlap and divergence in barriers identified before and after supervised PE implementation.

## Results:

### Participant characteristics.

The trainee group consisted of medical psychologists (comparable to clinical psychologists in the U.S.) and psychiatrists. Participants came from various provinces throughout Ukraine, with the highest representation from Kyiv region, followed by Lviv region, and smaller numbers from Kharkiv, Vinnytsia, Chernivtsi, Sumy, and Kropyvnytskyi regions. Most participants were female. Their professional backgrounds varied considerably, ranging from early-career professionals to those with moderate experience, as well as a substantial number with over ten years in the field (Table 2).

To assess whether participation in supervision improved trainees’ confidence in delivering PE interventions, we compared self-reported confidence levels before the first supervision session and after six months of supervision. The results of a one-tailed Wilcoxon signed-ranks test indicated that participants ‘ confidence significantly increased over time, Z = −1.67, p = .048. Examining Table 3, we observe the statistics for each of the models being tested to predict changes in clinicians’ confidence. The details are described narratively below.

### Model 1: Supervision Adherence as a Predictor of Confidence:

**Figure 1:**
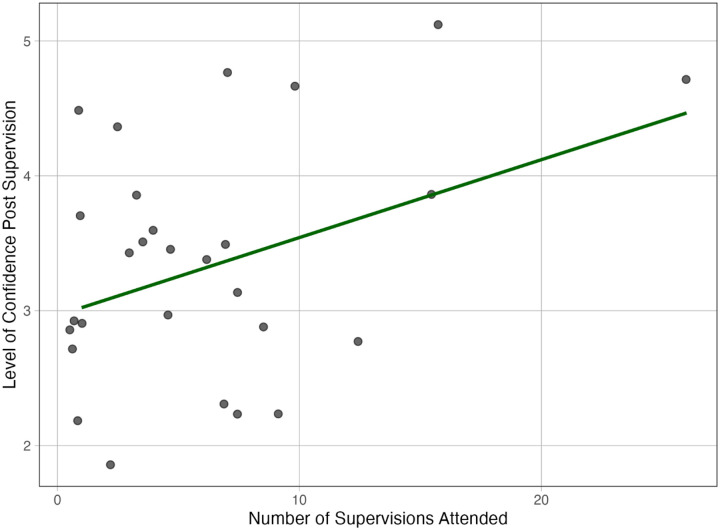


Results indicated that the number of supervision sessions attended was a significant predictor of post-supervision confidence (B = 0.059, SE = 0.023, β = .47, t = 2.54, p = .018). In contrast, pre-supervision confidence did not significantly predict post-confidence levels (B = −0.024, SE = 0.136, β = −.03, t = −0.17, p = .863). We evaluated model fit with the F-statistic and r-square. The overall model approached statistical significance F(2, 25) = 3.35, p = .051, and explained approximately 21.1% of the variance in post-supervision confidence (R^2^ = .21, Adjusted R^2^ = .15). Figure 1 illustrates the results of Model 1, showing a positive linear relationship between the number of supervision sessions attended and post-supervision confidence scores. The figure highlights that increased attendance was associated with higher confidence in implementing PE, supporting the hypothesis that regular supervision contributes significantly to skill development.

### Model 2: Case Experience as a Predictor of Confidence:

**Figure 2.**
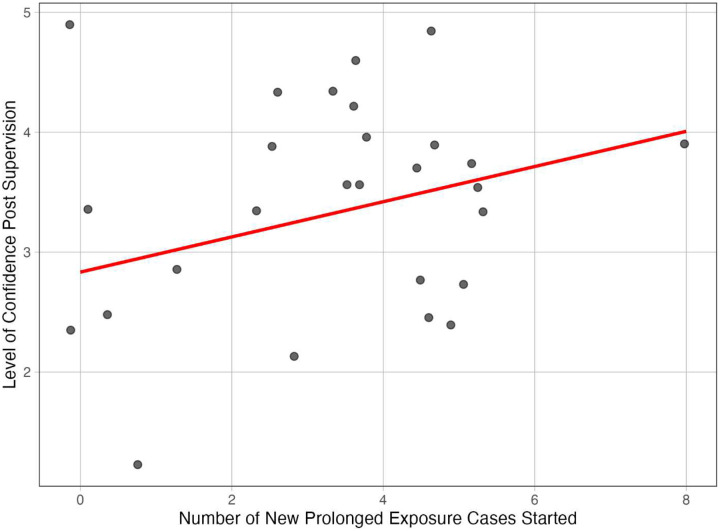


The number of new PE cases initiated was a significant positive predictor of post-supervision confidence (B = 0.16, SE = 0.07, β = .44, t = 2.33, p = .029). However, pre-supervision confidence was not a significant predictor (B = 0.13, SE = 0.15, β = .17, t = 0.88, p = .386). The overall model is trend-level significant F(2,24)=2.76, p=0.08, explaining a modest portion of variance (R2=0.019, Adjusted R^2^ = .12). Figure 2 displays the results of Model 2, demonstrating a positive association between the number of new PE cases initiated and post-supervision confidence. The trend suggests that direct clinical experience with PE cases contributes meaningfully to clinicians’ growing confidence, even after accounting for baseline confidence levels.

### Model 3: Combined Effects of Supervision and Experience:

Both the number of PE cases initiated (B = 0.14, SE=.06, β = .38, t=2.12, p = .045) and the number of supervision sessions attended (B = 0.05, CE=.02, β = .40, t=2.27, p = .033) were significant positive predictors of post-training confidence. Similar to Model 1 and 2, pre-training confidence was not a significant predictor (B = 0.06, 95% SE=.14, β = .07, t=.39, p = .701). The overall model was statistically significant (F(3, 23) = 3.87, p = .022). When considered together, the predictors explained 33.6% of the variance (R^2^ = .34, Adjusted R^2^ = .25). These results confirmed our prediction that both predictors had unique contributions to post-supervision confidence.

### Change of perception of barriers toward implementing evidence-based practices:

A thematic analysis was conducted to identify and compare perceived barriers to implementing evidence-based trauma care, specifically PE psychotherapy before and after six months of supervised clinical practice. The findings reflect a clear shift in perceived barriers from systemic and structural limitations to pragmatic, client-centered challenges as trainees progressed through clinical experience.

### Pre-Practice Thematic Findings:

Five major themes emerged from the pre-practice responses:

1. Lack of Trained Specialists (10 references). Many participants highlighted a shortage of qualified mental health professionals trained in evidence-based methods: “Not enough qualified specialists”
2. Knowledge and Training Gaps (9 references). Respondents frequently mentioned insufficient training and limited exposure to evidence-based care: *“Low quality education for psychologists or [training is] too expensive”*
3. Limited Access and Infrastructure (8 references). Participants noted logistical barriers, especially in underserved or rural areas: *“Not enough clinics that provide EB therapy”, “EB therapy available only in big cities”*
4. Cultural and Stigma-Related Barriers (7 references). Mental health stigma and distrust in psychotherapy were consistently cited: *“Negative beliefs against everything that starts with ‘psycho’”*
5. Contextual Challenges (6 references). The ongoing war was cited as a profound, omnipresent barrier: *“Many other problems in our society now, including war, it’s not the best time for psychological treatment”*

### Post-Practice Thematic Findings

**Figure 3.**
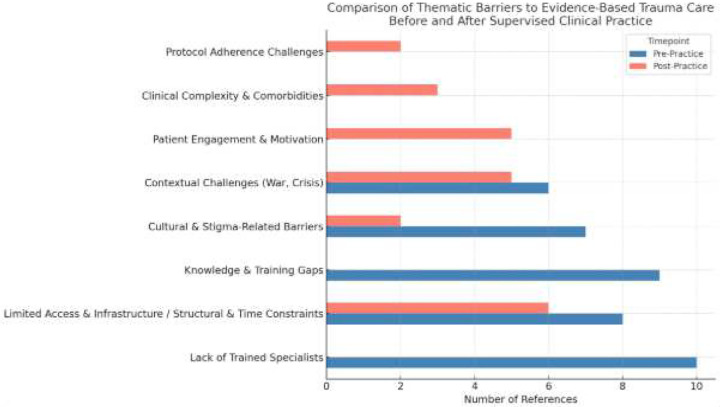


Following six months of supervised PE delivery, participants reported a notable shift in focus toward individual-level and treatment delivery barriers (Figure 3). Six major themes were identified:

1. Structural and Time Constraints (6 references). Respondents described difficulty scheduling and delivering full PE protocols, especially in military settings: *“They are given 21 days for psychological rehabilitation… then sent back to the front line”*
2. Patient Engagement and Motivation (5 references) Resistance, avoidance, or low motivation among patients emerged as significant impediments to treatment initiation and retention. *“Patients’ motivation is low, they are not willing to do the homework required by PE.”*
3. Impact of War and Military Context (5 references). Although less prominent than in pre-practice responses, war remained a notable contextual barrier: *“The continuation of hostilities has left both patients and clinicians exhausted, living under constant threat. This makes it extremely difficult to plan therapy sessions”, ” In the case of working with soldiers, a major challenge is the limited time available for psychological care before they return to military service.”*
4. Clinical Complexity and Comorbidities (3 references). Practitioners noted overlapping symptoms and comorbid diagnoses complicating implementation: *“Problem of blurring symptoms, misunderstanding, and combining with other symptoms (Traumatic Brain Injury)”*
5. Protocol Adherence Challenges (2 references). Participants shared struggles with delivering the PE protocol as designed: *“The therapist has limited time with the patient, which is insufficient for delivering Prolonged Exposure (PE) therapy effectively.”*
6. Stigma and Societal Perceptions (2 references). While less emphasized, stigma was still mentioned: *“My patients avoid seeking therapy due to fear of judgment or negative perceptions”*

## Discussion

Consistent with our initial hypothesis, trainees’ confidence in implementing PE therapy significantly increased after six months of weekly supervision delivered remotely by U.S.-based experts. Both the number of supervision sessions attended and the number of PE cases initiated during this period were significant predictors of post-training confidence. Together, these factors explained a larger proportion of the variance than either alone, underscoring the independent and additive contributions of structured supervision and hands-on clinical experience. In parallel, trainees’ perceptions of implementation barriers shifted notably. Pre-supervision responses emphasized systemic and educational gaps, such as lack of training or infrastructure, while post-supervision reflections focused on patient-level challenges, including engagement and motivation. Notably, “Patient Engagement,” a theme absent prior to supervision, emerged as a salient concern afterward, while “Knowledge Gaps” and “Lack of Specialists” diminished. These shifts suggest that supervised clinical practice not only builds confidence but also deepens awareness of the practical challenges of delivering PE in real-world settings, while removing barriers like “knowledge gaps”.

Distinct from models that focus primarily on administrative oversight or general skill development (Falender & Shafranske, 2008), our supervision emphasized fidelity to PE, case-based learning, and culturally responsive feedback. These components align with implementation science best practices, which highlight the need for sustained, model-specific support to ensure long-term adoption of evidence-based treatments (Beidas & Kendall, 2010; Fixsen et al., 2010). The structured, expert-led format likely facilitated skill development and protocol adherence, while culturally attuned supervision helped foster engagement and reduce resistance to implementation (Schoenwald et al., 2011). At the same time, our supervision model differed substantially from standard in-person, locally delivered clinical supervision described in the literature (Milne, 2007); (Falender & SHAFRANSKE, 2023). Conducted via telecommunication across time zones, it required adaptation to remote learning formats and asynchronous follow-up due to scheduling constraints, factors known to affect supervision quality and engagement (Falender et al., 2021). Unlike traditional settings, our supervisees were non-native English speakers, introducing additional complexity in communication and comprehension of clinical concepts, which can impact learning and the supervisory alliance (Goodyear et al., 2023). Regular attendance was also hindered by frequent power outages and unstable internet access, common in regions affected by conflict (Bank, 2023). The ongoing war in Ukraine created a persistent backdrop of psychological distress, setting this supervision apart from models implemented in peaceful, resource-stable environments. Additionally, our supervisees had very limited prior exposure to PE therapy, necessitating a steeper learning curve and more intensive support. Cultural differences in trauma narratives and emotional expression required supervisors to continuously adjust their style to ensure cultural responsiveness and effectiveness (Jobson & Dalgleish, 2014). These compounded challenges underscore the resilience and adaptability required in delivering supervision in challenging conditions and highlight the unique contextual demands of supporting mental health care in conflict-affected regions.

Confidence was selected as our key outcome due to its frequent use in psychotherapy research as a proxy for implementation readiness (Gonsalvez & Calvert, 2014). While not a direct measure of competence, confidence is a meaningful predictor of protocol adherence and treatment uptake (Taylor et al., 2012). Its limitations, such as reliance on self-report and susceptibility to emotional and contextual influences, are well documented (An et al., 2020; Falender et al., 2021). Nonetheless, in global mental health contexts where formal fidelity assessments may be impractical, confidence remains a pragmatic and informative indicator of training impact (Lohani & Sharma, 2023). Longitudinal assessment of confidence changes throughout supervision provides valuable information about the psychological readiness of therapists to implement challenging trauma-focused treatments independently. In trauma therapy, this is especially important given the emotionally intense nature of exposure work, where therapist hesitation or uncertainty can negatively affect client outcomes (Zoellner et al., 2011).

Despite significant logistical and contextual challenges, including conducting supervision in a non-native language, across time zones, and amid an active war, our findings demonstrate that structured, ongoing supervision is both feasible and effective in improving clinicians’ confidence to deliver PE. Notably, both the number of supervision sessions attended, and the number of PE cases initiated independently and jointly predicted post-supervision confidence, highlighting the importance of combining consistent supervision with practical clinical experience.

In the context of PE therapy, case load tracking provides insight into trainees’ willingness to initiate trauma-focused treatment with eligible clients, a decision that often reflects confidence and competence. Treatment completion rates are particularly informative, as premature termination in exposure therapies may indicate therapist difficulty managing client distress or maintaining appropriate protocol delivery (Lutz et al., 2022).

Despite formidable barriers, including active conflict, limited infrastructure, and linguistic and cultural differences, our findings demonstrate that high-quality, remote supervision can be both feasible and impactful. The improvement in clinician confidence and the reframing of perceived barriers highlight the potential of targeted supervision models to support mental health care delivery in even the most challenging contexts. These findings underscore the importance of investing in adaptable supervision infrastructure, especially in global mental health and post-conflict recovery initiatives.

## Limitations

Several limitations should be considered when interpreting the findings of this study. First, the sample size was relatively small, which limits the statistical power and generalizability of the results. Additionally, supervision and training were conducted in English, a second language for all trainees, which may have affected comprehension and communication despite participants’ functional fluency. The remote nature of supervision, conducted across multiple time zones, introduced further logistical challenges, such as scheduling constraints, asynchronous communication, and frequent power outages due to ongoing conflict in Ukraine. These factors may have impacted both the consistency of attendance and the quality of engagement in supervision sessions. Moreover, the use of a single self-report item assessing therapist confidence, which, while commonly used as a proximal indicator in implementation science, does not capture objective clinical competence or treatment fidelity. The absence of additional outcome measures, such as observed adherence to the PE protocol or client outcomes, limits the depth of interpretation regarding the effectiveness of supervision. Additionally, the lack of a control or comparison group prevents causal inferences, and the observed changes cannot be definitively attributed to the supervision intervention alone. Although this study contributes to the literature on clinical supervision in crisis-affected contexts, the model of supervision implemented here differed in several key respects from standard models described in the literature, most notably in its delivery format, cultural and linguistic context, and the high level of external stressors. These differences limit the comparability of our findings with existing studies and highlight the need for further research to explore how supervision functions across diverse global mental health settings.

## Conclusion

Although confidence is an imperfect proxy for clinical competence, it remains a pragmatic and meaningful indicator in global mental health contexts where direct observation is not always feasible. Our findings align with and extend further current supervision literature, demonstrating that fidelity-focused, culturally responsive, and context-sensitive supervision models can be adapted to low-resource or crisis settings without sacrificing effectiveness. Our findings have important implications for mental health systems aiming to build sustainable workforce capacity. By equipping clinicians with the tools, support, and supervision needed to deliver high-quality trauma-focused care, training models such as the one described here can contribute to long-term improvements in the quality of life for individuals with PTSD. Scalable supervision infrastructures, especially those that integrate remote delivery, cultural adaptation, and experiential learning, offer a viable path for increasing access to evidence-based interventions in underserved or conflict-affected regions. Strengthening local clinician capacity through supervision not only enhances individual therapist performance but also lays the foundation for broader system-level resilience and recovery in post-trauma communities.

## Figures and Tables

**Table 1.** Content of PE training.

EBC	Instructional Time	Curriculum
Prolonged Exposure	16 hours	Introduction & Psychoeducation
Therapy	(4 session, 4-hours each)	Establishing Trust/Safety
		Breathing & Relaxation Training
		Imaginal Exposure
		In Vivo Exposure
		Processing & Reflection
		Generalization
		Relapse Prevention

**Table 2:** 

Demographic of Interest		N (%)
Format	Online	29 (100.0%)
Sex	Female	18 (62.07%)
	Male	11 (37.93%)
Profession	Psychology	17 (58.62%)
	Psychiatry	5 (17.24%)
	Psychiatry with additional training in Psychology	7 (24.14%)
*Background	Clinical	29 (100%)
	Academic	17 (58.62%)
	Military	4 (13.79%)
Years of experience	1–2 years	4 (13.79%)
	3–5 years	6 (20.69%)
	6–10 years	8 (27.59%)
	10+	11 (37.93%)
Geographic region	Kyiv	11 (37.93%)
	Lviv	4 (13.79%)
	Kharkiv	3 (10.34%)
	Vinnytsia	2 (6.90%)
	Chernivtsi	2 (6.90%)
	Kropyvnytskyi	1 (3.45%)
	Sumy	1 (3.45%)
	Poltava	1 (3.45%)
	Ternopil	1 (3.45%)
	Ivano-Frankivsk	1 (3.45%)
	Khmelnytskyi	1 (3.45%)
	Kherson	1 (3.45%)

* Overlap within groups

**Table 3:** Regression Results Predicting Post-Supervision Confidence Across Three Models

Model	Variable	B	SE	t	P
Model 1	# of sessions attended	0.059	0.023	2.54	.018
	Pre-training confidence	−0.024	0.136	−0.17	.863
Model 2	# of cases initiated	0.160	0.070	2.33	.029
	Pre-training confidence	0.130	0.150	0.88	.386
Model 3	# of sessions attended	0.050	0.020	2.27	.033
	# of cases initiated	0.140	0.060	2.12	.045
	Pre-training confidence	0.060	0.140	0.39	.701

## Data Availability

Data is available upon request. Please contact the corresponding author.

## Availability of data and materials:

The datasets generated and/or analyzed during the current study are available from the corresponding author upon reasonable request.

## List of abbreviations

PE: Prolonged Exposure
EBC: Evidence-Based Care
PTSD: Post Traumatic Stress Disorder
QA/QC: Quality Assurance/Quality Control
